# Thick airway surface liquid volume and weak mucin expression in pendrin-deficient human airway epithelia

**DOI:** 10.14814/phy2.12480

**Published:** 2015-08-04

**Authors:** Hyun Jae Lee, Jee Eun Yoo, Wan Namkung, Hyung-Ju Cho, Kyubo Kim, Joo Wan Kang, Joo-Heon Yoon, Jae Young Choi

**Affiliations:** 1 Brain Korea 21 PLUS Project for Medical Science, Yonsei UniversitySeoul, Korea; 2 Research Center for Human Natural Defense System, Yonsei UniversitySeoul, Korea; 3 Department of Otorhinolaryngology, Yonsei UniversitySeoul, Korea; 4 Airway Mucus Institute, Yonsei University College of MedicineSeoul, Korea; 5 Department of Otorhinolaryngology-Head and Neck Surgery, Kangdong Sacred Heart Hospital, Hallym University College of MedicineSeoul, Korea; 6 College of Pharmacy, Yonsei Institute of Pharmaceutical Sciences Yonsei UniversityIncheon, Korea

**Keywords:** anion channel, asthma, SLC26A4

## Abstract

Pendrin is an anion exchanger whose mutations are known to cause hearing loss. However, recent data support the linkage between pendrin expression and airway diseases, such as asthma. To evaluate the role of pendrin in the regulation of the airway surface liquid (ASL) volume and mucin expression, we investigated the function and expression of pendrin and ion channels and anion exchangers. Human nasal epithelial cells were cultured from 16 deaf patients carrying pendrin mutations (DFNB4) and 17 controls. The cells were treated with IL-13 to induce mucus hypersecretion. Airway surface liquid thickness was measured and real-time polymerase chain reaction was performed targeting various transporters and *MUC5AC*. Anion exchanger activity was measured using a pH-sensitive fluorescent probe. Periodic acid-Schiff staining was performed on the cultured cells and inferior turbinate tissues. The ASL layer of the nasal epithelia from DFNB4 subjects was thicker than the controls, and the difference became more prominent following IL-13 stimulation. There was no difference in anion exchange activity after IL-13 treatment in the cells from DFNB4 patients, while it increased in the controls. Goblet cell metaplasia induced by IL-13 treatment seen in the controls was not observed in the DFNB4 cells. Furthermore, the periodic acid-Schiff staining-positive area was lesser in the inferior turbinate tissues from DFNB4 patients that those from controls. Pendrin plays a critical role in ASL volume regulation and mucin expression as pendrin-deficient airway epithelial cells are refractory to stimulation with IL-13. Specific blockers targeting pendrin in the airways may therefore have therapeutic potential in the treatment of allergic airway diseases.

## Introduction

Pendrin, encoded by SLC26A4, is a membrane protein that exchanges anions such as HCO_3_^−^ and I^−^ for Cl^−^ (Mount and Romero 2004). The protein is predominantly expressed in the inner ear, thyroid, and kidney (Scott et al. 1999; Royaux et al. 2000, 2001; Lacroix et al. 2001). Pendrin presumably acts as an HCO_3_^−^/Cl^−^ exchanger in the inner ear, and mutations cause prelingual hearing loss with enlarged vestibular aqueducts (DFNB4) (Yang et al. 2005; Albert et al. 2006; Nakaya et al. 2007). In the thyroid, pendrin is essential for I^−^ uptake, and defects in this protein may cause goiter and possibly hypothyroidism. Goiter combined with hearing loss is known as Pendred syndrome (Morgans and Trotter 1958; Reardon et al. 1999).

Pendrin is also expressed in the airway epithelia (Kuperman et al. 2005; Pedemonte et al. 2007; Nakagami et al. 2008; Nakao et al. 2008; Di Valentin et al. 2009; Nofziger et al. 2011) and recent data suggest that pendrin expression is associated with airway diseases. Pendrin expression is upregulated in airway tissues of patients with asthma and allergic rhinitis (Kuperman et al. 2005; Lewis et al. 2008; Nakagami et al. 2008; Nakao et al. 2008; Di Valentin et al. 2009; Izuhara et al. 2009). Moreover, recent study showed that patients harboring bi-allelic *SLC26A4* mutations do not experience asthma (Nakao et al. 2008), although the prevalence of asthma among these patients is not significantly lower compared to controls due to the low numbers of subjects. Animal studies also showed the role of pendrin in the pathogenesis of asthma. Ovalbumin-induced airway hyperresponsiveness and inflammation are attenuated in pendrin-knockout mice (Nakagami et al. 2008; Madeo et al. 2009) and the overexpression of pendrin in airway epithelia of mice resulted in increased airway hyperresponsiveness (Nakagami et al. 2008). These findings suggest that pendrin may play an important role in the development of asthma.

Homeostasis of the volume and composition of the airway surface liquid (ASL) is essential for maintaining the mucociliary clearance system (Tarran et al. 2001; Boucher 2003). Airway surface liquid volume is regulated by the coordinated interactions of various ion transporters, such as epithelial sodium channels (ENaC) and cystic fibrosis transmembrane conductance regulators (CFTR). A recent report (Nakagami et al. 2008) indicates that pendrin is involved in ASL volume regulation. ASL thickness was shown to increase following allergic cytokine, interleukin (IL)-13, stimulation in the airway epithelia of pendrin-knockout mice. Another interesting discovery is that pendrin may strongly participate in the secretion of airway mucus. Overexpression of pendrin induces mucus hypersecretion in mice, and pendrin has been shown to be a key modulator of IL-13-induced mucus secretion (Nakao et al. 2008).

Based on these recent findings, pendrin may be a potential new drug target for asthma and other inflammatory airway diseases. However, before deciding on the value of pendrin as a new drug target, further elucidation of the physiological role of pendrin in human airways is necessary because the inner ear and the thyroid phenotypes of pendrin-knockout mice have shown to be different from those of patients harboring SLC26A4 mutations and the physiology underlying ASL homeostasis and mucus secretion in human airways is quite different from that in animal models.

In this study, we compare the ASL thickness, mucin expression, and the response to stimulation with IL-13 in nasal epithelial cells cultured from patients with *SLC26A4* mutations (DFNB4) to those in controls.

## Materials and Methods

### Subjects and tissue harvesting

This study was approved by the Institutional Review Board of the Yonsei University Health System. Human nasal mucosa was obtained from the inferior turbinates of 16 deaf patients harboring bi-allelic *SLC26A4* mutations (DFNB4) during cochlear implantation. Nasal mucosa was also harvested in 17 controls who underwent septoplasty for correction of deviated nasal septum. These controls had no history of allergic rhinitis or chronic sinusitis, and a genetic study confirmed they did not carry any *SLC26A4* mutations. A genetic analysis confirmed the presence of the bi-allelic *SLC26A4* mutation in all 16 deaf patients. The genotypes of the DFNB4 patients were as follows: H723R homozygote (*n* = 6), H723R/IVS7-2A>G (*n* = 6), IVS7-2A>G homozygote (*n* = 2), IVS7-2A>G/M147V (*n* = 1) and IVS7-2A>G/I302K (*n* = 1). H723R, M147V, I302K mutations produce a misfolding pendrin which retains within the ER. Slicing mutation, IVS7-2A>G, result in complete deletion of exon 8. Therefore, all the mutations in this study cannot produce a functioning pendrin. Table1 shows the characteristics of the subjects.

**Table 1:** Subject characteristics

	N	Age	Male [no. (%)]
DFNB4*	16	18 ± 12	8 (50)
Control	17	25 ± 7	10 (59)

* Patients with bi-allelic mutation in *SLC26A4*.

### Cell culture and IL-13 treatment

Primary cultures of human nasal epithelial (HNE) cells were obtained as previously described (Yoon et al. 1999). Passage-2 cells were seeded into a Transwell-Clear culture insert with a 0.45-*μ*m pore size (Costar Co., Cambridge, MA) at a density of 2 × 10^5^ cells/cm^2^. The cells were maintained in a 1:1 mixture of Dulbecco’s modified Eagle’s medium (Lonza, Walkersville, MD) and bronchial epithelial growth medium (Lonza), supplemented with the following growth factors according to the manufacturer’s instructions: hydrocortisone (0.5 *μ*g/mL), insulin (5 *μ*g/mL), transferrin (10 *μ*g/mL), epinephrine (0.5 *μ*g/mL), triiodothyronine (6.5 *μ*g/mL), gentamicin (50 *μ*g/mL), amphotericin B (50 *μ*g/mL), retinoic acid (15 ng/mL), bovine pituitary extract (50 *μ*g/mL), bovine serum albumin (1.5 *μ*g/mL), and epidermal growth factor (0.5 ng/mL). The cells were maintained submerged for the first 7 days, after which they were exposed to the apical air interface for the remainder of the culture period. The cells were used between 14 and 21 days after the establishment of the air-liquid interface. At all stages of culture, the cells were maintained at 37°C under 5% CO_2_ in an air incubator. To stimulate the cells, they were treated basolaterally with 10 ng/mL of recombinant human IL-13 (R&D Systems, Minneapolis, MN) for 7 days.

### Quantitative real-time PCR

Total RNA was isolated from primary cultures of HNE cells from five patients harboring bi-allelic *SCL26A4* mutations and four controls. RNA was isolated using the TRIzol reagent (Invitrogen, San Diego, CA) according to the manufacturer’s protocol. Purified RNA samples were reverse transcribed with a cDNA Synthesis Kit (Applied Biosystems, Foster City, CA) according to the manufacturer’s instructions. Quantitative Real-time PCR for pendrin, epithelial sodium channel (ENaC) *α*, *β*, *γ*, CFTR, ANO-1, Anion exchanger (AE) 2, AE3, AE4 and SLC26A3, SLC26A6, SLC26A7, SLC26A8, SLC26A9, SLC26A11, MUC5AC was performed using the 7300 Real-Time PCR System (Applied Biosystems) and the DyNAmoHSSYBR Green qPCR Kit (Finnzymes, Espoo, Finland). The primers employed for real time PCR are listed in Table S1.

### Measurement of ASL thickness

The thickness of the ASL was measured in HNE cells using a modified version of the method described by Terran et al. (Tarran et al. 2005). Briefly, the cells were washed, and 20 *μ*L of PBS containing 0.2% (v/v) Texas Red-dextran (Invitrogen) was added onto the apical cell surface to label the ASL layer. A 100-*μ*L aliquot of perfluorocarbon (Fluorinert FC-770; 3M, St. Paul, MN) was then added to the apical surface to prevent evaporation of the ASL. After 12 h, the cultures were transferred to the stage of an inverted confocal microscope (LSM 700; Carl Zeiss MicroImaging Inc., Thornwood, NY) and the height of the ASL was measured at five predetermined points in the cultures (one central, four circumferential) via XZ scans. Images were reconstructed three dimensionally and analyzed using Imaris 7.1 software (Bitplane Co, Zurich, Switzerland).

### Short circuit current

Passage-2 primary cultured human nasal epithelial cells were mounted in Ussing chambers (Physiologic Instruments, San Diego, CA). Amiloride, CFTRinh-172, ATP were added to the apical solution, and an equal volume of vehicle was added at the same time to the basolateral solution. Symmetrical HCO_3_^−^-buffered solutions (120 mmol/L NaCl, 5 mmol/L KCl, 1 mmol/L MgCl_2_, 1 mmol/L CaCl_2_, 10 mmol/L d-glucose, 5 mmol/L HEPES, and 25 mmol/L NaHCO_3_, pH 7.4) were used. Cells were bathed for a 10 min stabilization period and aerated with 95% O_2_/5% CO_2_ at 37°C or room temperature. Short circuit current was measured using an EVC4000 MultiChannel V/I Clamp (World Precision Instruments, Sarasota, FL) and recorded using PowerLab/8sp (AD Instruments, Castle Hill, Australia).

### Measurement of anion exchange activity

Measurements of the pH_i_ in the HNE cells were performed with a pH-sensitive fluorescent probe, bis-carboxyethyl-carboxyfluorescein (BCECF) (Invitrogen, San Diego, CA), as described previously (Yoon et al. 2008). Briefly, the measurements were performed in PBS- or IL-13-treated cells, in which a cluster of cells showing green fluorescent protein (GFP) fluorescence was exposed to BCECF, and the pH_i_ was monitored. After adding BCECF, the cells were perfused with HCO_3_^−^-buffered solution (120 NaCl, 5 KCl, 1 MgCl_2_, 1 CaCl_2_, 10 d-glucose, 5 HEPES and 25 NaHCO_3_ in mmol/L, pH 7.4), and BCECF fluorescence was recorded at excitation wavelengths of 490 nm and 440 nm at a resolution of 2 sec on a recording setup (Delta Ram; PTI Inc., Birmingham, NJ). The Cl^−^/HCO_3_^−^ exchange activities were estimated from the initial rate of the pH_i_ increase as a result of Cl^−^ removal in the HCO_3_^−^-containing buffer (25 mmol/L HCO_3_^−^ with 5% CO_2_). The intrinsic buffer capacity (*β*_i_) was measured as described previously (Yoon et al. 2008).

### Histology and Periodic acid-Schiff (PAS) staining

Periodic acid-Schiff (PAS) staining was performed with a PAS staining kit (Sigma, St. Louis, MO) according to the manufacturer’s protocol. The harvested nasal mucosa and HNE cells on the transwell inserts were gently washed with PBS and fixed with 4% paraformaldehyde. After deparaffinization and hydration, the slides were immersed in periodic acid solution for 5 min at room temperature. After being rinsed in distilled water, the slides were immersed in Schiff’s reagent for 15 min at room temperature, and then washed in running tap water for 5 min. Slides were counterstained in Hematoxylin Solution for 90 sec and then rinsed in running tap water, dehydrated, cleared, and permanently mounted. For morphometric analysis, images of the PAS-stained slides (five different slides of tissue from each patient) were acquired with a Leica DM750 LED microscope and PAS-positive cells were counted.

### Statistics

Statistical analysis was performed with SPSS software for PC, version 19 (SPSS Inc., Chicago, IL). Differences between groups regarding anion exchange activities and the rates of pendrin positivity were evaluated via one-way analysis of variance. A *P*-value of less than 0.05 was considered statistically significant.

## Results

### ASL thickness; DFNB4 patients versus controls

**Figure 1:**
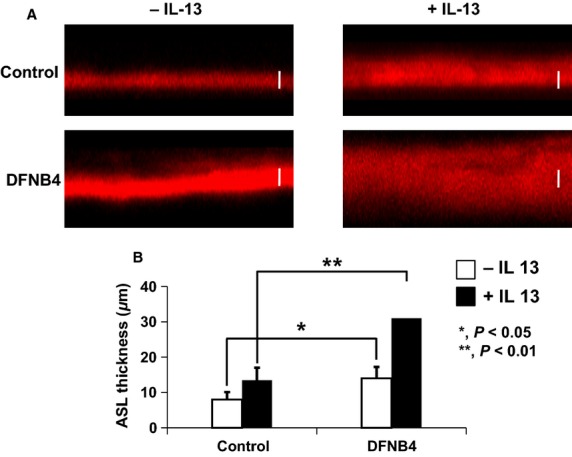
Airway surface liquid (ASL) thickness in human nasal epithelia from patients harboring *SLC26A4* mutations (DFNB4) and controls. Confocal images (A) and summary of ASL thickness measurements (B) at 12 h after the apical addition of 20 *μ*L of PBS containing Texas Red-dextran to primary nasal epithelial cultures from DFNB4 patients (*n* = 5) and controls (*n* = 4) in the absence or presence of IL-13 (10 ng/mL). Scale bars, 10 *μ*m. *P < 0.05, **P < 0.01, compared with the controls. Error bars, S.E.

First, we measured the ASL thickness in HNE cells from patients harboring *SLC26A4* mutations (DFNB4) and controls. Twelve hours after PBS/Texas Red-dextran loading, the ASL thickness in the DFNB4 patients (14.1 ± 2.9 *μ*m) was thicker than that in the controls (8.0 ± 2.1 *μ*m, *P* < 0.05). The increase in ASL thickness following IL-13 stimulation was more prominent in DFNB4 cells (31.3 ± 7.6 *μ*m) than in controls (13. 5 ± 2.8 *μ*m) (Fig.1).

### Molecular and functional expression of Na^+^ and Cl^−^ channels

**Figure 2:**
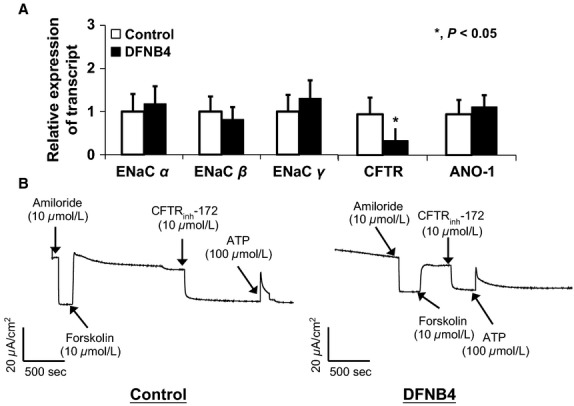
Expression of epithelial sodium channels (ENaC) and Cl^−^ channels (CFTR and ANO-1) in human nasal epithelial cells from patients harboring *SLC26A4* mutations (DFNB4) and controls. (A) The mRNA expression patterns in the cells from patients with DFNB4 (*n* = 3) are similar to those in controls (*n* = 3), except for CFTR, which shows low levels of expression in the epithelia from DFNB4 patients. *indicates *P* < 0.05, compared to controls. (B) Amiloride-sensitive and ATP-induced short circuit current is similar in the cells from patients with DFNB4 (*n* = 4) to those in the controls (*n* = 3). Forskolin–induced current is smaller in the DNFB cells than those in the control cells.

We compared the mRNA expression patterns of ion channels involved in ASL volume regulation to rule out the possibility that the difference in ASL thickness between the pendrin mutants and controls stemmed from the differential expression of these channels. The expression patterns of ENaC subtypes *α*, *β*, and *γ* and ANO-1 were similar between DFNB4 patients and controls. However, CFTR showed lower expression in the DFNB4 patients (0.36 ± 0.25-fold of that in the controls) (Fig.2A).

We also measured short circuit current (Isc) in airway epithelial cells. The amplitude of the amiloride-sensitive (22.1 ± 7.2 *μ*A/cm^2^) and ATP-sensitive current (31.1 ± 6.7 *μ*A/cm^2^) in DFNB4 cells, which reflects ENaC and ANO-1 activity, respectively, were not significantly different between disease controls (28.1 ± 9.4 *μ*A/cm^2^ and 32.8 ± 7.8 *μ*A/cm^2^) However, the forskolin-induced current, which indicates CFTR activity, was smaller in DFNB cells (29.4 ± 9.2 *μ*A/cm^2^)than in disease controls (42.1 ± 6.6 *μ*A/cm^2^) (Fig.2B)**.**

### Changes of channel and pendrin expressions in response to IL-13

**Figure 3:**
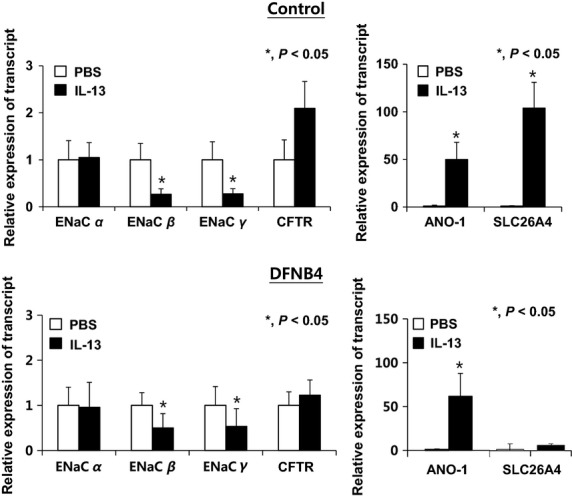
Changes in ion channel and pendrin expression in response to IL-13 treatment (10 ng/ml). In both the control (*n* = 3) and *SLC26A4* mutant (DFNB4) (*n* = 3) nasal epithelia, ENaC*β* and *γ* expression are suppressed, and ANO-1 expression is greatly increased (by more than 50 fold) following IL-13 treatment. In the control epithelia, pendrin is also upregulated (approximately 100 fold) following IL-13 treatment. *indicates *P* < 0.05.

When the HNE cells from controls were treated with IL-13, ENaC subtypes *β* and *γ* were downregulated, whereas ANO-1 (49 ± 18 fold of PBS) was upregulated. As previously reported, pendrin expression was also greatly increased by 104 ± 27 fold following treatment with IL-13. The response of the pendrin-deficient epithelial cells to IL-13 treatment was similar to the response in the controls: ENaC subtypes *β* and *γ* were downregulated, and ANO-1 was upregulated (61 ± 26 fold) (Fig.3).

### Anion exchanger expression and activity

**Figure 4:**
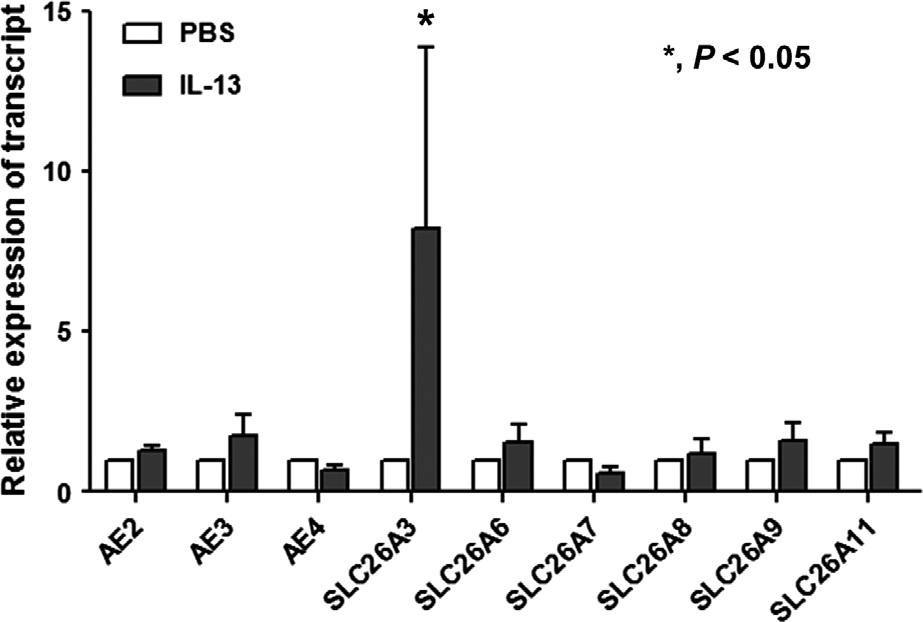
Anion exchanger expression in cultured human nasal epithelia from controls. Along with pendrin, various anion exchangers (*AE2, AE3 and AE4*) and *SLC26A* family transporters (*SLC26A3, SLC26A6, SLC26A7, SLC26A8, SLC26A9,* and *SLC26A11*) are expressed in nasal epithelia from controls (*n* = 5). Only *SLC26A3* is increased by IL-13 (10 ng/mL). *indicates *P* < 0.05 compared with the PBS-treated cells.

**Figure 5:**
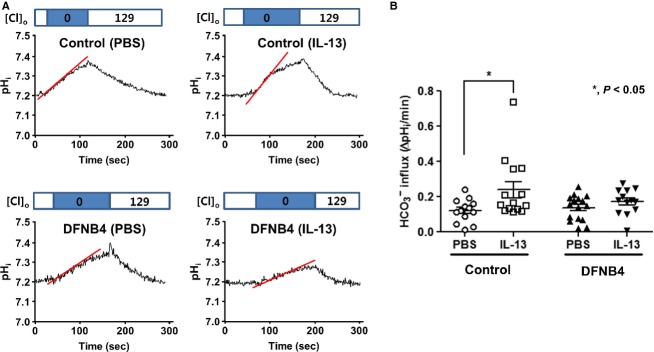
Anion exchange activity in human nasal epithelia cultured from patients with *SLC26A4* mutations (DFNB4) and controls. (A) Representative tracings of anion exchange activity. (B) Measurements of anion exchange activity showing significantly increased activity following IL-13 treatment (10 ng/mL) in the controls (*n *=* *4) but not in epithelia from patients harboring *SLC26A4* mutations (DFNB4, *n* = 5). *indicates *P* < 0.05 compared with the PBS-treated cells.

In addition to *SLC26A4*, various anion exchangers, including *AE2, AE3, AE4, SLC26A3, SLC26A6, SLC26A7, SLC26A8, SLC26A9,* and *SLC26A11*, were found to be expressed in the HNE cells of the controls. When the cells were treated with IL-13, *SLC26A3* was upregulated (by 8.2 ± 5.4 fold compared to PBS treatment) along with *SLC26A4*. The other anion exchangers did not respond to IL-13 stimulation (Fig.4). We also examined anion channel activity by measuring the Cl^−^/HCO_3_^−^ exchange ratio. The anion exchange activity in epithelia from the patients harboring *SLC26A4* mutations (DFNB4) (0.12 ± 0.07 ΔpH/min) was not different from that in the controls (0.15 ± 0.06 ΔpH/min) under basal conditions. When the HNE cells from the controls were treated with IL-13 for 7 days, the anion channel activity was significantly increased (0.23 ± 0.17 ΔpH/min, *P* < 0.05), but not in the epithelia from patients with *SLC26A4* mutations (0.18 ± 0.06 ΔpH/min) (Fig.5). The molecular and functional data indicate that pendrin is a major anion exchanger that responds to IL-13 and that the intracellular and ASL pH may be regulated by various anion exchangers other than pendrin under basal conditions.

### *MUC5AC* expression and goblet cell differentiation

**Figure 6:**
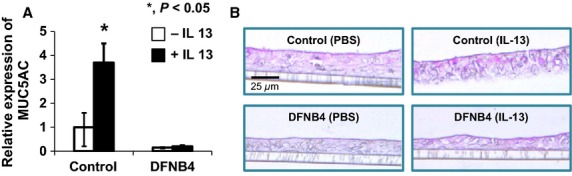
*MUC5AC* expression and goblet cell differentiation in cultured human nasal epithelial cells. (A) Real-time PCR analysis showing upregulation of *MUC5AC* by IL-13 (10 ng/ml) in controls (*n* = 3). In contrast, *MUC5AC* is weakly expressed and does not respond to IL-13 in epithelia from patients carrying *SLC26A4* mutations (DFNB4, *n* = 3). *indicates *P* < 0.05. (B) PAS staining showing goblet cell hyperplasia in nasal epithelial cells from controls following IL-13 treatment (10 ng/mL) for 7 days, but not in epithelia from DFNB4 patients. Scale bar represents 25 *μ*m.

**Figure 7:**
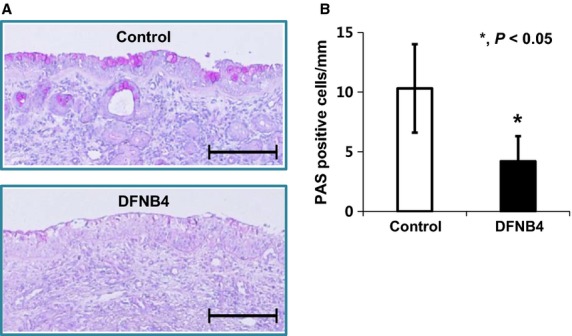
PAS staining of inferior turbinates. (A) Representative images shows a much lower amount of PAS staining in turbinate tissue from patients harboring *SLC26A4* mutations (DFNB4) compared with the tissues from controls (bars indicate 200 *μ*m) (B) Quantitative analysis of the abundance of PAS-positive cells in 6 DFNB4 and 6 controls. *indicates *P* < 0.05.

We also examined the role of pendrin in mucus secretion in the airway epithelia. Quantitative real-time PCR data showed that the expression of *MUC5AC,* a goblet cell marker, was greatly increased by IL-13 treatment in cultured cells from controls. In contrast, *MUC5AC* expression was comparatively low in cultured cells from DFNB4 patients, and it was not upregulated by IL-13 treatment (Fig.6A). Periodic acid-Schiff staining revealed goblet cell hyperplasia in the control HNE cells following IL-13 treatment, but this was not observed in the epithelia from DFNB4 subjects (Fig.6B). Histologic examination of the inferior turbinates also revealed that there was a much lower number of PAS-positive cells (3.7 ± 2.1/mm) in the nasal mucosa from DFNB4 patients compared with that from controls (10.3 ± 3.7/mm) (Fig.7).

## Discussion

A recent report (Nakagami et al. 2008) indicates that pendrin is involved in the regulation of ASL thickness in mice. Although the ASL thickness did not differ in cultured tracheal epithelial cells between wild-type and pendrin-deficient mice under basal conditions, the ASL became much thicker in pendrin-deficient mice compared to the cells from wild-type mice after stimulation with an allergic cytokine (Nakagami et al. 2008). However, pendrin regulates the pH of the ASL but has little effect on fluid secretion in Calu-3 cells, which are human serous airway epithelial cells (Garnett et al. 2011). Therefore, it was unclear whether pendrin regulates ASL volume in airway epithelia in humans. In our data, the expression pattern of ion transporters in airway epithelia was similar between the patients with *SLC26A4* mutations and controls except for the weak expression of CFTR in patients with *SLC26A4* mutations. However, the ASL layer was thicker in HNE cells from patients with *SLC26A4* mutations compared to controls. These data indicate that pendrin is involved in ASL volume regulation even in basal conditions. This finding is not consistent with data from the mouse airway. This discrepancy may stem from species differences in the role of ion transporters in airway epithelia. For instance, the expression patterns of ion channels are quite different between mice and humans as CFTR expression is barely detectable in mouse airway epithelia. Another possible reason for the conflicting results between reports may be attributable to a methodological problem. Nakagami et al. (2008) measured ASL thickness 3 min after PBS/dye loading, before ASL thickness had reached a steady state.

**Figure 8:**
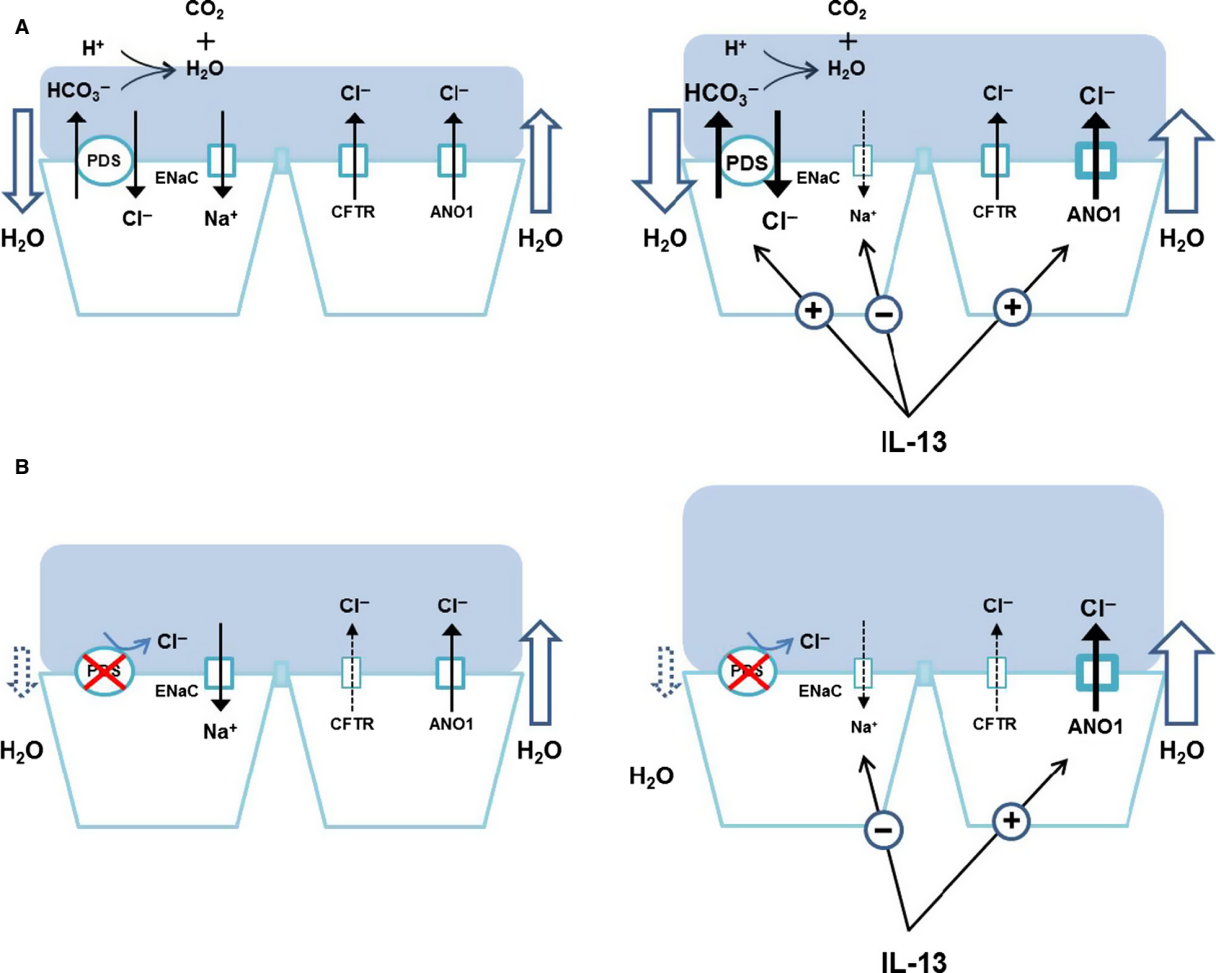
The hypothetical role of pendrin in the regulation of airway surface liquid (ASL). (A) Pendrin regulates ASL volume via Cl^−^ and subsequent water absorption. The secreted HCO_3_^−^ will evaporate, thus leading to net anion movement into the cells. Under inflammatory conditions, upregulated pendrin compensates for the over-secreted fluid by upregulated ANO-1 channels. (B) In pendrin-deficient airways, ASL thickness is increased due to the lack of Cl^−^ absorption by pendrin under basal conditions and cannot compensate for the over-accumulated fluid.

The mechanism by which pendrin regulates ASL is puzzling because this electroneutral transporter cannot generate an osmotic gradient. One possible mechanism underlying this phenomenon is the evaporation of secreted HCO_3_^−^, which results in net anion movement into the cell and subsequent Na^+^ absorption and generation of osmotic pressure for fluid absorption (Fig.8A). Interestingly, the difference of ASL height between normal and pendrin-deficient airway epithelia became much more prominent with IL-13 treatment. IL-13 induces airway fluid secretion and as a result, the ASL thickness is increased. In normal airway epithelial cells, the upregulation of pendrin (∼ 100 fold) following IL-13 treatment compensates for the thickened ASL via Cl^−^ absorption. However, presumably since this compensatory mechanism does not exist in the pendrin mutants, it results in an overaccumulation of ASL (Fig.8B).

Pendrin seems to be not only involved in ASL regulation, but also mucus secretion. Enforced expression of pendrin in NCI-H292 cells or mouse airway epithelia induces the upregulation of *MUC5AC* (Nakao et al. 2008). However, pendrin-deficient mice exhibit impaired airway hyperresponsiveness and eosinophilic inflammation, but not mucus production. Our data showed that *MUC5AC* was expressed at very low levels in the nasal epithelia from *SLC26A4* mutants and was not upregulated by IL-13 treatment, which contrasts with our findings in the cells of the controls. We also revealed that the goblet cell differentiation in basal condition and hyperplasia induced by IL-13 was minimal in the epithelia from patients with *SLC26A4* mutations. Furthermore, we demonstrated that the proportion of goblet cells in the nasal mucosa from patients with SLC26A4 mutation is much lower compared to that in controls. These data all together indicate that pendrin is a major participant in mucous cell differentiation and hyperplasia induced by allergic cytokines in airway epithelia. Although the exact role of pendrin in mucus secretion in the airways epithelia is unclear, pendrin may be involved in the infiltration of Th2 inflammatory cells which produce several inflammatory mediators such as EGF and TNF-*α*. These mediators can regulate the transcription of the mucin gene via activation of the transcription factors such as NF-*κ* B, as suggested by Rose & Voynow (Rose and Voynow 2006).

An interesting phenomenon is the linkage between the pathogenesis of asthma and pendrin. Pendrin knockout mice show less of an allergic response to OVA stimulation than their wild-type counterparts (Nakagami et al. 2008). Pendrin expression is upregulated after IL-4 stimulation in cultured airway epithelia (Pedemonte et al. 2007) and disease tissues, such as those from asthma, COPD, and rhinitis patients (Kuperman et al. 2005; Lewis et al. 2008; Ishida et al. 2012). Moreover, patients with pendrin mutations had a tendency to be resistant to asthma (Nakao et al. 2008). Based on our data, we postulate that the increase in ASL thickness observed in the airways of pendrin-deficient patients under basal conditions may decrease the chance of allergens coming into contact with the airway epithelia, and therefore hinder the initiation of allergic sensitization. Moreover, the dramatic increase in ASL thickness induced by allergic cytokines could further protect the airway epithelia against exposure to allergens in pendrin-deficient airways. Another possible mechanism explaining the lower incidence of asthma in patients harboring pendrin mutations is a defect in mucus secretion, although the exact molecular mechanism underlying this process is unclear.

One limitation of our study is that we used cultured nasal epithelia to represent the airways. Cultured nasal epithelia are not ideal model for studying the pathogenesis of asthma because the cellular phenotype and expression pattern of ion transporters of nasal epithelia is somewhat different from those of tracheobronchial epithelial cells. However, following the guidelines of our institutional review board, harvesting tracheobronchial cells from patients undergoing ear surgery is too invasive of a procedure, while it is much easier to obtain nasal mucosa.

The decreased expression of CFTR in DFNB4 patients is an interesting finding. Our preliminary coimmunoprecipitating experiment shows that immune complexes of airway epithelia lysate and CFTR antibody are clearly reactive with the pendrin antibody, which suggests that pendrin and CFTR may be coupled in chloride recycling. The possible functional coupling of pendrin and CFTR may become an important issue in the homeostasis of airway surface liquid. We need further experiments to investigate the exact mechanism of this coupling.

In summary, we showed that the ASL layer was increased in human airway epithelial cells from patients with *SLC26A4* mutation compared to controls, and this phenomenon was more prominent in allergic conditions. Pendrin seems to be essential in mucin expression and goblet cell development. These findings may explain the low incidence of allergic airway diseases in patients with *SLC26A4* mutation. Specific blockers targeting pendrin in the airway epithelium may represent promising candidate drugs for the treatment of allergic airway diseases such as asthma and allergic rhinitis.

## Conflict of interest

None declared.

## Supporting Information

**Table S1.** Primer sequences used for quantitative RT-PCR.
