# Supplementary material for: Thick airway surface liquid volume and weak mucin expression in pendrin-deficient human airway epithelia
Source: Physiol Rep. 2015 Aug 4;3(8):e12480. doi: 10.14814/phy2.12480 (PMC4562566; doi:10.14814/phy2.12480)
Supplement: Supplementary file 1 [file phy20003-e12480-sd1.docx]

Table S1. Primer sequences used for quantitative RT-PCR

| SLC26A3 | 5'- GAG AGCACAGGAGGC AAA AC -3'  5'- CAG CAA ACT GCATCAGCATT-3' |
| --- | --- |
| SLC26A4 | 5'- GTGACGTCATTTCGG GAG TT-3' 5'- GCCATG CTC AGA ACAACA GA-3' |
| SLC26A6 | 5'- AGA AGCAGGAGCAGCTGA AG-3'  5'- CCTTCAGTGTGGACC CAT CT-3' |
| SLC26A7 | 5'- TGCACC CAG TGTTTGGTT TA-3'  5'- ACGTGGATCCTTTGC ATT TC-3' |
| SLC26A8 | 5'- GCT GAG ACC GAG ACCAAG AC -3'  5'- GACTGGGTGGAAGCC ATA GA -3' |
| SLC26A9 | 5'- GGATTCGAACCAGGA GAT GA -3' 5'- GAT CAG GGCTCC TAG CAC AG -3' |
| SLC26A11 | 5'- CTT CAT TTC CTA CCC CGT CA -3'  5'- TACCCT GGT CTC TGCAAT CC -3' |
| **SLC4A2**  **(AE2)** | 5'- GCTGGG AGA GAAGACACAGG -3'  5'- CAG GTGGTTGCTGCTACA GA -3' |
| **SLC4A3**  **(AE3)** | 5'- AGCCCTATGTGACCAAGG TG -3'  5'- TACTCATCCTGGCCATCCTC -3' |
| **SLC4A9**  **(AE4)** | 5'- AGGCCA AAA GAC AGA GAC GA -3' 5'- AGA AGCGGCTGGTCTTTA CA -3' |
| MUC5AC | 5'- CAGCCACGTCCCCTTCAATA -3  5'- ACCGCATTTGGGCATCC -3' |
| CFTR | 5'- AGG AGG CAG TCT GTC CTG AA -3  5'- CAC TGC TGG TAT GCT CTC CA -3 |
| ANO-1 | 5'- ACG AGG AGT GTG TGA AGA GG -3  5'- TGC ATG GTC CCG TTC TTA CT -3 |
| ENaC α | 5'- CAG CCC ATA CCA GGT CTC AT -3  5'- ATG GTG GTG TTG TTG CAG AA -3 |
| ENaC β | 5'- TCC TAC CCT CGT CCC TAC CT -3  5'- CCA GGA AGG AGA AAA CCA CA -3 |
| ENaC γ | 5'- ACC ACC AGC CAT GGT CTA AG -3  5'- GTT CAG GTC CCG GGA TTT AT -3 |
